# Abnormal fermentations in table-olive processing: microbial origin and sensory evaluation

**DOI:** 10.3389/fmicb.2013.00091

**Published:** 2013-05-10

**Authors:** Barbara Lanza

**Affiliations:** Consiglio per la Ricerca e la sperimentazione in Agricoltura, Centro di Ricerca per l’Olivicoltura e l’Industria Olearia, Città Sant’AngeloPescara, Italy

**Keywords:** table olives, lactic acid bacteria, yeast, bacteriophages (phages), starter cultures, abnormal fermentation, sensory analysis

## Abstract

The process of transformation of table olives from tree to table is the result of complex biochemical reactions that are determined by the interactions between the indigenous microflora of the olives, together with a variety of contaminating microrganisms from different sources [fiber-glass fermenters, polyvinyl chloride (PVC) tanks, pipelines, pumps, and water], with the compositional characteristics of the fruit. One of the most important aspects of improving the quality of table olives is the use of selected microorganisms to drive the fermentation. These can supplant the indigenous microflora and, in particular, the complementary microflora that are responsible for spoilage of canned olives. In this context, from a technological point of view, a well-characterized collection of microrganisms (lactic acid bacteria, yeast) that can be isolated from the matrix to be processed (the olive fruit) will provide the basis for the development of starter culture systems. These cultures can be fully compatible with the typical products and will guarantee high quality standards. Inoculation of the brine with such selected starter cultures will reduce the probability of spoilage, and help to achieve an improved and more predictable fermentation process. Control of the fermentation processes can thus occur through chemical, chemico-physical and microbiological approaches, and since 2008, also through organoleptic evaluation (*COI/OT/MO/Doc. No 1*. *Method for the sensory analysis of table olives)*. This last has established the necessary criteria and procedures for sensory analysis of the negative, gustatory and kinaesthetic sensations of table olives, which can also be attributed to abnormal proliferation of microrganisms. It also sets out the system for commercial classification, through assessment of the median of the defect predominantly perceived.

## INTRODUCTION

The production of fermented foods is one of the oldest biotechnologies known to mankind. In particular, the fermentation of vegetables, which is a practice that originated in the Orient, has been used as a means of preserving food for more than 2,000 years. Table olives (*Olea europaea* L.) are fermented products that are widely diffuse (Garrido Fernandez et al., 1997). The most important production zone of table olives is the Mediterranean area, although olives are consumed on a large scale all over the world. Indeed, their consumption is also expanding, due to the increasing popularity of the Mediterranean diet.

Olives are picked at different stages of maturity, and they are then processed to eliminate the characteristic bitterness caused by their oleuropein glucoside, and thus to make them suitable for human consumption. There are several ways to prepare table olives, but the most widespread methods are known as “treated green olives in brine in the Seville or Spanish style” (Garrido Fernandez et al., 1997; Sánchez Gómez et al., 2006) and “natural black olives directly placed in brine in the Greek style” (Balatsouras, 1990).

## MAIN MICROBIAL ALTERATIONS DURING TABLE-OLIVE PROCESSING

Briefly, the Seville or Spanish system consists of alkaline treatment with NaOH or lye (1.5–4.5°Bé) to remove the bitterness of the olive fruit, which is following by water washing to eliminate the residual lye, and then fermentation in brine to enhance the nutritional and sensory characteristics of the fruit. After the alkaline treatment, the pH of the olive flesh reaches 11.0–13.0, which is reduced to 8.0–9.0 after repeated washing.

After the washing, the olive fruit are immersed in 6–10% NaCl solution (brine). Spontaneous fermentation starts as soon as the olives are in the brine. In the first phase of fermentation, when the Gram-negative bacteria prevail, the pH decreases from 8.0 to 9.0 to about 6.0. This phase lasts until the development of lactic acid bacteria (normally after 48–72 h). Most of the microorganisms that develop in this first phase are Gram-negative (*Enterobacter*, *Citrobacter*, *Aeromonas*, *Escherichia*, and *Klebsiella*). If the decrease in the pH during the first few days of fermentation is not fast enough, deterioration of the olives can quickly set in, due to Enterobacteriaceae and other microbial groups that can reach high cell densities and form “gas pockets,” resulting in softening and breakage of the cuticle, and other defects.

**FIGURE 1:**
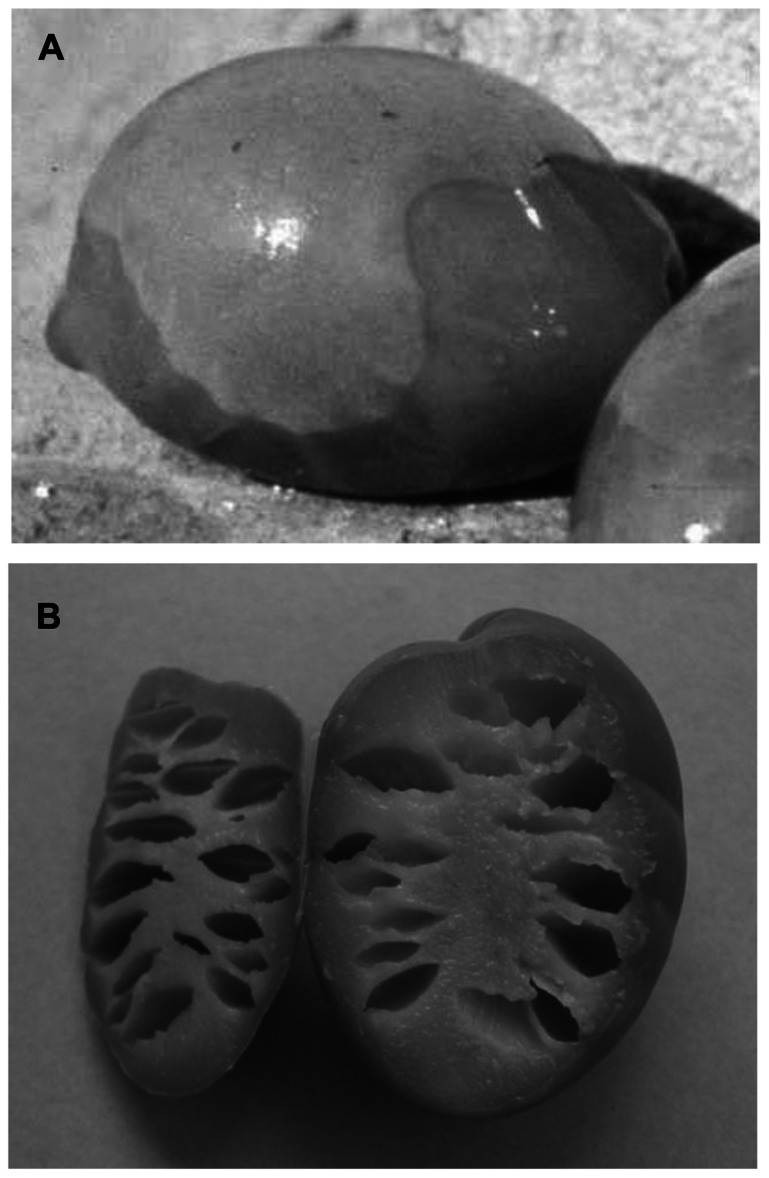
**Olive fruit showing hypocuticular (A) and intramesocarpic (B) gas-pockets**.

During the first phase of the fermentation process, there are frequent cases where these gas-generating Gram-negative bacteria can take over, which consume the sugars and release CO_2_. This CO_2_ can accumulate as pockets of gas below the epidermis (hypocuticular gas pockets) or within the pulp itself (intramesocarpic gas pockets; **Figure 1**). The olives affected by these changes appear to show bubbles on their surface, which is known as “fish-eye” (Vaughn et al., 1972), or as a narrow belt, which is known as “olive gated” or “alambrado” (Gililland and Vaughn, 1946; Borbolla y Alcalá et al., 1959, Borbolla y Alcalá et al., 1960). High pH can also contribute to the development of *Clostridium*, which results in fermentation that is termed putrid (reminiscent of the smell of decomposing organic matter) or butyric (reminiscent of the smell of rancid butter). This malodorous fermentation caused by butyric anaerobes such as *Clostridium butyricum* produces olives that are completely fissured (Gililland and Vaughn, 1946). The gas evolved reduces the density of the fruit, which then float on the surface. This process can also lead to the appearance of olives without gas pockets, due to the development of the pulp cavity, which sometimes extends through the core, and which is due to the accumulation of CO_2_ from the respiration of olive tissue and the activity of certain microorganisms that release CO_2_ as a normal product of their metabolism (**Table 1**). To reduce the appearance of this defect, an aerobic fermentation system has been developed, in which air is blown into the container (the fermenter) where the processing takes place (Borbolla y Alcalá et al., 1960; Garrido Fernandez et al., 1987; Marsilio et al., 2008a,b). This system removes the CO_2_, which induces a change in the predominant microflora in the process, with particular reference to yeast. This treatment thus supports the aerobes with so-called oxidative metabolism (which transform nutrients in the presence of oxygen) at the expense of those of fermentation (metabolism that takes place in the absence of oxygen).

**Table 1:** The main microbial alterations during table-olive processing. Red, defects that are detectable by sensory analysis.

Spoilage	Microorganism responsible	Reference
Gas-pockets (fish-eye and alambrado)	*Saccharomyces kluyveri*	Duran Quintana et al. (1979)
	*Saccharomyces cerevisiae*	Vaughn et al. (1972)
	*Pichia anomala*	Borbolla y Alcalá et al. (1959)
	*Enterobacter sp.*	Borbolla y Alcalá et al. (1960)
	*Citrobacter sp.*	Gililland and Vaughn (1946)
	*Aeromonas sp.*	
	*Escherichia sp.*	
	*Klebsiella sp.*	
	*Clostridium butyricum*	
Putrid fermentation	*Desulfovibrio aestuarii*	Levin and Vaughn (1966)
Butyric fermentation	*Clostridium butyricum*	Gililland and Vaughn (1946)
	*Clostridium beijerinckii*	
	*Clostridium fallax*	
	*Clostridium acetobutylicum*	
Zapateria	*Propionibacterium pentosaceum*	Gonzalez Cancho et al. (1980)
	*Propionibacterium zeae*	Gonzalez Cancho et al. (1973)
	*Propionibacterium acnes*	Plastourgos and Vaughn (1957)
	*Clostridium sporogenes*	Kawatomari and Vaughn (1956)
	*Clostridium bifermentans*	
Musty	*Penicillium crustosum*	Marsilio and Spotti (1987)
	*Penicillium digitatum*	
	*Penicillium roquefortii*	
	*Penicillium simplicissimum*	
	*Penicillium aurantiogriseum*	
	*Penicillium expansum*	
	*Penicillium herquei*	
	*Penicillium viridicatum*	
	*Aspergillus niger*	
	*Alternaria alternata*	
Winey-vinegary	Yeast with alcoholic metabolism	Borbolla y Alcalá et al. (1958)
	*Acetic bacteria*	
Yeast or white spots	*Lactobacillus plantarum*	Samish and Dimant (1963)
		Vaughn et al. (1953)
Botulism	*Clostridium botulinum*	Cawthorne et al. (2005)
		Fenicia et al. (1992)
Softening	*Saccharomyces cerevisiae*	Golomb et al. (2013)
	*Pichia anomala*	Arroyo-López et al. (2012)
	*Saccharomyces kluyveri*	Vaughn et al. (1972)
	*Pichia manshurica*	Vaughn et al. (1969)
	*Pichia kudriavzevii*	Balatsouras and Vaughn (1958)
	*Candida boidinii*	Nortje and Vaughn (1953)
	*Rhodotorula minuta* var.	
	*minuta*	
	*Rhodotorula rubra*	
	*Rhodotorula glutinis *var.	
	*glutinis*	
	*Penicillium sp.*	
	*Fusarium sp.*	
	*Aspergillus niger*	
	*Bacillus pumilus*	
	*Bacillus subtilis*	
Killer activity	*Debaryomyces hansenii*	Marquina et al. (1997)
	*Kluyveromyces marxianus*	Hernández et al. (2008)
	*Pichia anomala*	
	*Pichia guilliermondii*	
	*Saccharomyces cerevisiae*	
Phage infection	*Bacteriophages of lactic acid bacteria*	Lanza et al. (2012)

The development of pectinolytic (*Saccharomyces oleaginosus*, *S. kluyveri*, *Hansenula anomala*, *Pichia manshurica*, *Pichia kudriavzevii*, *Candida boidinii*, *Rhodotorula minuta*, *R. rubra*, *Rhodotorula glutinis*, *Aspergillus niger*, *Penicillium* sp. and *Fusarium* sp.) and cellulolytic (*Cellulomonas *sp.) yeast and moulds is associated with “softening” of the fruit. This is due to the action of their degrading enzymes that, respectively, act on pectic substances that form the middle lamella, which leads to cell separation, and act on cellulose, hemicellulose and polysaccharides, which damages the cell walls (Vaughn et al., 1969; Vaughn et al., 1972; Arroyo-López et al., 2012; Golomb et al., 2013). The softening of the fruits is also associated with the presence of *Bacillus* and Gram-negative organisms that are normally present in this phase (Nortje and Vaughn, 1953)

A second phase begins when the pH reaches about 6.0, and this stage lasts until the development of lactobacilli (normally 2 weeks). During this phase, the Gram-negative microorganisms progressively decrease, until they disappear altogether. Reducing sugars and glucosides, the basic sources of carbon needed for the development of lactobacilli and other microorganisms, pass from the olive flesh into the brine, where they are used by heterofermentative or homofermentative microorganisms that transform them into lactic acid.

Most of the microorganisms that grow in this second phase are lactococci, within the genus *Pediococcus* (homofermentative strain) and *Leuconostoc* (heterofermentative strain). These produce lactic acid, which contributes to the further lowering of the pH. This then favors the growth of lactobacilli (in the third phase) that are aciduric, with their optimal growth between pH 5.5 and 5.8. This phase is characterized by abundant growth of homofermentative lactobacilli, with a predominance of *Lactobacillus plantarum*. One of the most common abnormalities associated with olives treated with the Seville style is known in the industry as “yeast or white spots.” These are small white spots that can develop between the skin and the flesh of the olive. Microscopic and microbiological studies have shown that this defect is due to *L. plantarum* bacteria colonies, rather than to yeast (Vaughn et al., 1953; Samish and Dimant, 1963; Garrido Fernandez et al., 1997; Kailis and Harris, 2007).

A population of yeast with fermentative metabolism can then co-exist with the lactic acid bacteria. If these yeast do not become prevalent, they are not considered to be harmful to the process, and indeed, they can help to enhance the sensory properties of the finished product (through alcoholic fermentation). The presence of yeast and their role in table-olive processing (including their potential role as starter cultures) has been discusses by several authors (Psani and Kotzekidou, 2006; Arroyo-López et al., 2008; Nisiotou et al., 2010). The main yeast species that are usually found in table-olive processing are: *Wickerhamomyces anomalus*, *Saccharomyces cerevisiae*, *Pichia membranifaciens*, *Kluyveromyces lactis*, *Debaryomyces hansenii*, *Pichia anomala*, *Pichia guilliermondii*. Some of those yeast can secrete pore-forming proteinaceous killer toxins that are active against other sensitive (killer-sensitive) yeast strains. Since the first description of the killer factor of *S. cerevisiae*, many halotolerant killer yeast, which show killer activity in the presence of NaCl, have been isolated from many fermented foods, like miso, soy sauce, and salted vegetables. These include *D. hansenii* and *P. anomala*. Hernández et al. (2008) reported that *Debaryomyces* was the genus with the highest percentages of killer strains tested, and they suggested that starter cultures of these isolates can be used as a biocontrol method against spoilage yeast. In other studies that have been performed with yeast isolated from spontaneous fermentation of olive brines, strains of the genera *Pichia*, *Kluyveromyces*, *Candida*, and *Torulaspora* also show killer activity against killer-sensitive strains of *S. cerevisiae* (Marquina et al., 1997).

At the end of the lactic fermentation, the pH decreases to < 4.0, and thus the acidity increases, which ensures the preservation of the product. The lactic fermentation ends when the supply of available carbohydrates is exhausted (e.g., glucose from glucosides and reducing sugars). At the end of this third phase, if the product is not pasteurized, then during storage it might undergo further unwanted fermentation that can see the development of the genus *Propionibacterium*, which can metabolize the lactic acid, to produce acetic acid and propionic acid. This potential fourth phase is characterized by an increase in pH and volatile acidity, and a decrease in lactic acid. Also, the neo-formation of a volatile fatty acid, cyclohexanecarboxylic acid (Montano et al., 1992, 1996) and the production of biogenic amines, such as cadaverine and tyramine, (Garcia et al., 2004), might be related to “zapateria” of the olives. These conditions also encourage the development of *Clostridium *(*C. sporogenes*, *C. bifermentans*), which together with *Propionibacterium* (*Propionibacterium pentosaceum*, *Propionibacterium Zeae*, *Propionibacterium acnes*) can promote zapatera spoilage (Kawatomari and Vaughn, 1956; Plastourgos and Vaughn, 1957; Gonzalez Cancho et al., 1973, Gonzalez Cancho et al., 1980). To avoid the uncontrolled and harmful development of these bacteria, the pH should be kept < 4.0 and the NaCl in the brine should be raised to >8%.

An increase in pH and anaerobic conditions of packaged olives can also promote the growth of *C. botulinum* (Fenicia et al., 1992; Cawthorne et al., 2005). This intoxication is common in olives that are darkened by oxidation. These olives are also known as Californian-style black olives, ripe or semi-ripe olives, or simply black olives. Here, the pH is between 5.8 and 7.9, and the NaCl is between 1 and 3%. Due to these chemical characteristics, which do not guarantee the safety of the product, the olives are darkened by oxidation and have to be sterilized to prevent any possibility of growth of foodborne pathogens.

The Greek system for olive processing is the best known method for “natural olives.” To obtain naturally fermented olives, the fruit are placed directly into brine (6–10% NaCl, usually), in which the fermentation takes place. The elimination of oleuropein is very slow and incomplete; thus, the final product is slightly bitter, but very tasty. In the Greek-style process the hydrolysis of oleuropein is attributed to the enzymatic reactions of the indigenous microorganisms, through their β-glucosidase and esterase (Ciafardini et al., 1994; Marsilio et al., 1996; Marsilio and Lanza, 1998). In such spontaneous fermentations, the natural microflora present on the fruit skin is often uncontrollable and unpredictable. The spontaneous fermentation of Greek-style olives mainly depends on the domination of the brine by lactic acid bacteria, which are mainly represented by *L. plantarum*, and yeast.

After the debittering and washing steps, olives treated according to the Spanish style have a pH between 8.0 and 9.0, whereas freshly picked olives processed by the Greek style are immersed in brine at a pH between 5.0 and 6.5 (Panagou et al., 2003; Marsilio et al., 2005, 2006). To accelerate the fermentation process, it would be useful to select and use starter cultures of lactic acid bacteria that will develop and/or promote fermentation at different pHs. Several studies have been carried out to evaluate the technological functionality of selected lactic acid bacteria and enterococci in Spanish-style green-olive processing. *Enterococcus casseliflavus* and *L. pentosus *have been proposed as starter cultures to accelerate lactic acid formation at pH 9 (immediately after washing; Sanchez et al., 2001; de Castro et al., 2002). In this case, obviously, the strain used as the starter does not need to be oleuropeinolytic, because lye has previously been used to remove the bitter glucoside. Thus, the importance of this type of starter is to reduce the lag phase and the risk of spoilage (Ruiz-Barba et al., 1994; Vega Leal-Sanchez et al., 2003; Bevilacqua et al., 2010).

Secoiridoid glucosides (oleuropein, demethyl-oleuropein, ligstroside) and other β-glucosides are the principal fermentative substrates in the olive fruit. These are enzymatically hydrolysed by β-glucosidase (E.C.3.2.1.21), which releases glucose and aglycones. The aglycones can then be completely degraded by esterases (E.C. 3.1.1.1., E.C. 3.1.1.2.) into simple and non-bitter phenolics, such as hydroxytyrosol, tyrosol, and elenolic acid. Some strains of *L. plantarum* can produce β-glucosidase and esterase and use the glucose from the β-glucosides as a source of carbon. Therefore, these strains can hydrolyse oleuropein and other bitter glucosides, which contributes to the debittering process. The degradation of oleuropein is evaluated by inoculation of the strains to be tested in MRS broth without glucose and with the addition of oleuropein, with analysis for the degradation products (hydroxytyrosol, aglycones) by gas chromatography (Ciafardini et al., 1994) or high performance liquid chromatography (HPLC; Landete et al., 2010; Zago et al., 2013). The evolution of individual phenolic compounds upon processing of the Ascolana cv. olive fruit without and with lactobacilli as an inoculant has been described previously (Marsilio et al., 2006). In that study, the degradation rate of oleuropein was faster in the presence of the starter inoculants, with only trace levels after 15 days of fermentation, thereby defining a significant role of lactobacilli in olive debittering. Similarly, decreases in oleuropein and increases in hydrotyrosol have been shown, which reduced the debittering phase to 8 days during controlled fermentation of Leccino cv. olives using *L. pentosus* 1MO as a starter culture (Servili et al., 2006). Six *L. plantarum* strains studied by Zago et al. (2013) showed a high degree of oleuropein degradation after 24 h, and this glucoside completely disappeared after a week. These results were confirmed by hydroxytyrosol accumulation. In particular, strains Lp793, B51, and Lp994 showed the highest degrees of oleuropein degradation and hydroxytyrosol accumulation. Other studies have isolated and selected oleuropeinolytic lactic acid bacteria from fermenting Moroccan green olives, including *L. plantarum*, *L. pentosus*, *L. brevis*, and *Pediococcus pentosaceus* (Ghabbour et al., 2011).

## PHAGE ATTACK

The presence of bacteriophages in the brine of table olives might be a cause for failure of the acidification process carried out by the lactic acid bacteria. This can have a more or less serious impact on the technological process and the final product characteristics (e.g., abnormal fermentation, inhibition of starter culture). A phage infection in the processing of table olives can be the real obstacle to the use of starter cultures as inoculum (Lanza et al., 2012). In addition to the safeguarding of traditional products, mixed starters or natural brine that are used as “mothers” are generally not very sensitive to phages as the complex microbial composition provides a kind of self-defense. This is because different strains can sustain an attack by a phage without serious repercussions on the fermentation (resistant strains prevail, while the sensitive strains are lysed). The bacteriophages are also a useful tool for the typing of strains. Phages can provide a sort of natural selection across strains with different phage sensitivities, by helping to regulate the development of these microbial ecosystems. Indeed, a protocol previously described for dairy starters was adapted in our laboratory to search for the presence of viral particles in brine. This involves: (a) incubation for 24 h of strains used as starters in MRS-Ca^2+^ broth medium; (b) addition of an aliquot of the brine to be tested (in this case the same brine as that used for fermentation, suitably cleaned of microbial cells through centrifugation and sterile filtration). The clarification of the cultures after the appropriate incubation can be considered as indicative of bacterial lysis. For the enumeration of the bacteriophages, the plaque-based assay is used. This assay is carried out in petri dishes with a monolayer of the host cells that are then infected with an aliquot of brine with the bacteriophage at varying dilutions; these are then covered with a semi-solid medium. A viral plaque is formed when a virus infects a cell within the fixed cell monolayer. The virus-infected cell will lyse and spread the infection to adjacent cells where the infection-to-lysis cycle is repeated. The infected cell area will create a plaque (an area of infection surrounded by uninfected cells) which can be seen by eye or under an optical microscope. These plaques are used to calculate the number of plaque forming units per sample unit volume (i.e., pfu/mL). Direct observations of phage attacks can be performed using transmission electron microscopy (Bradley, 1967), scanning electron microscopy (Lanza, 2011), epifluorescence, and atomic force microscopy (Zago et al., 2012).

## SENSORY EVALUATION OF TABLE OLIVES

Fermentation processes can be controlled through chemical, chemico-physical and microbiological approaches, and since 2008 in particular, this has also involved organoleptic evaluation (*COI/OT/MO/Doc. No 1*. *Method for the sensory analysis of table olives)*. On 25 November, 2011, following Decision No DEC-18/99-V/2011, the International Olive Council adopted a revised version of the method of sensory evaluation (COI/OT/MO No 1/Rev. 2). This is applicable solely to the fruit of the cultivated olive tree (*O. europaea *L.) that has been suitably treated or processed, and has been prepared for trade or for final consumption as table olives, in accordance with the trade standards applying to table olives (COI/OT/NC No 1, 2004). This method established the necessary criteria and procedures for sensory analysis of table olives, including for negative gustatory and kinaesthetic sensations. It also defined the system for the commercial classification of table olives. This classification includes the median of the defect predominantly perceived; i.e., the defect that is perceived at the greatest intensity. The defects that are attributable to the abnormal proliferation of microrganisms are: putrid and butyric fermentations, zapateria, and musty and winey-vinegary flavors. Putrid and butyric fermentations and zapateria are discussed above. Musty defects depend on mould attacks during the processing of the olives. The main microorganisms responsible for mustiness are *Penicillium* (*P. crustosum*, *P. digitatum*, *P. roqueforti*, *P. simplicissimum*, *P. aurantiogriseum*, *P. expansum*, *P. herquei*, *P. viridicatum*), *A. niger *and *Alternaria alternata *(Marsilio and Spotti, 1987). Winey-vinegar sensations are associated with the production of ethanol, CO_2_, acetic acid, and organic acids through alcoholic fermentation, which is principally associated with the development of yeast and acetic bacteria (sensations reminiscent of wine or vinegar). The intensities of these bitter and acid tastes are also evaluable. The bitter sensation depends on the presence of bitter substances that come from the fruit, which are mainly polyphenols. This might therefore be more intense in preparations in which the debittering is incomplete due to the actions of the natural olive microorganisms. An acid sensation defines the taste associated with acids that are naturally present in the flesh of the olive fruit (e.g., tartaric acid, malic acid, and citric acid) or that are produced during the lactic fermentation by homofermentative and heterofermentative lactic acid bacteria (e.g., lactic acid, acetic acid). The acidity might also depend on inappropriate use of acids as correctives for acidity (e.g., citric acid). High levels of acid sensation are also found in olives that have been prepared with the addition of vinegar (e.g., Kalamata olives). Finally, through this sensory evaluation, it is possible to define the level of softening of the fruit, which corresponds to the low levels of the kinaesthetic sensation of hardness. The main microbial alterations in table olives that are detectable by sensory analysis are shown in **Table 1**.

## CONCLUSION

Transformation of table olives from the tree to the table is the result of complex biochemical reactions that depend on interactions between the indigenous microflora of the olives, together with a variety of contaminating microrganisms from different sources (fiber-glass fermenters, PVC tanks, pipelines, pumps, and water), with the compositional characteristics of the fruit. One of the most important aspects for the improvement of the quality of table olives would be the use of selected microorganisms to drive the required fermentation. These would also supplant the indigenous microflora and those that are responsible for spoilage of canned olives. In this context, a well-characterized collection of microrganisms (lactic acid bacteria, yeast), possibly isolated from the matrix to be processed (the olive fruit), would provide the basis for the development of starter cultures, while remaining fully compatible with the typical products, to guarantee the maintenance of high quality standards. Finally, a phage-database is a natural and necessary complement of a collection of lactic acid bacteria of technological interest.

## Conflict of Interest Statement

The author declares that the research was conducted in the absence of any commercial or financial relationships that could be construed as a potential conflict of interest.
